# Supplementary material for: Cloning of maize chitinase 1 gene and its expression in genetically transformed rice to confer resistance against rice blast caused by Pyricularia oryzae
Source: PLoS One. 2024 Jan 16;19(1):e0291939. doi: 10.1371/journal.pone.0291939 (PMC10791007; doi:10.1371/journal.pone.0291939)
Supplement: S1 Raw images — (PPTX) [file pone.0291939.s001.pptx]

## Slide 1
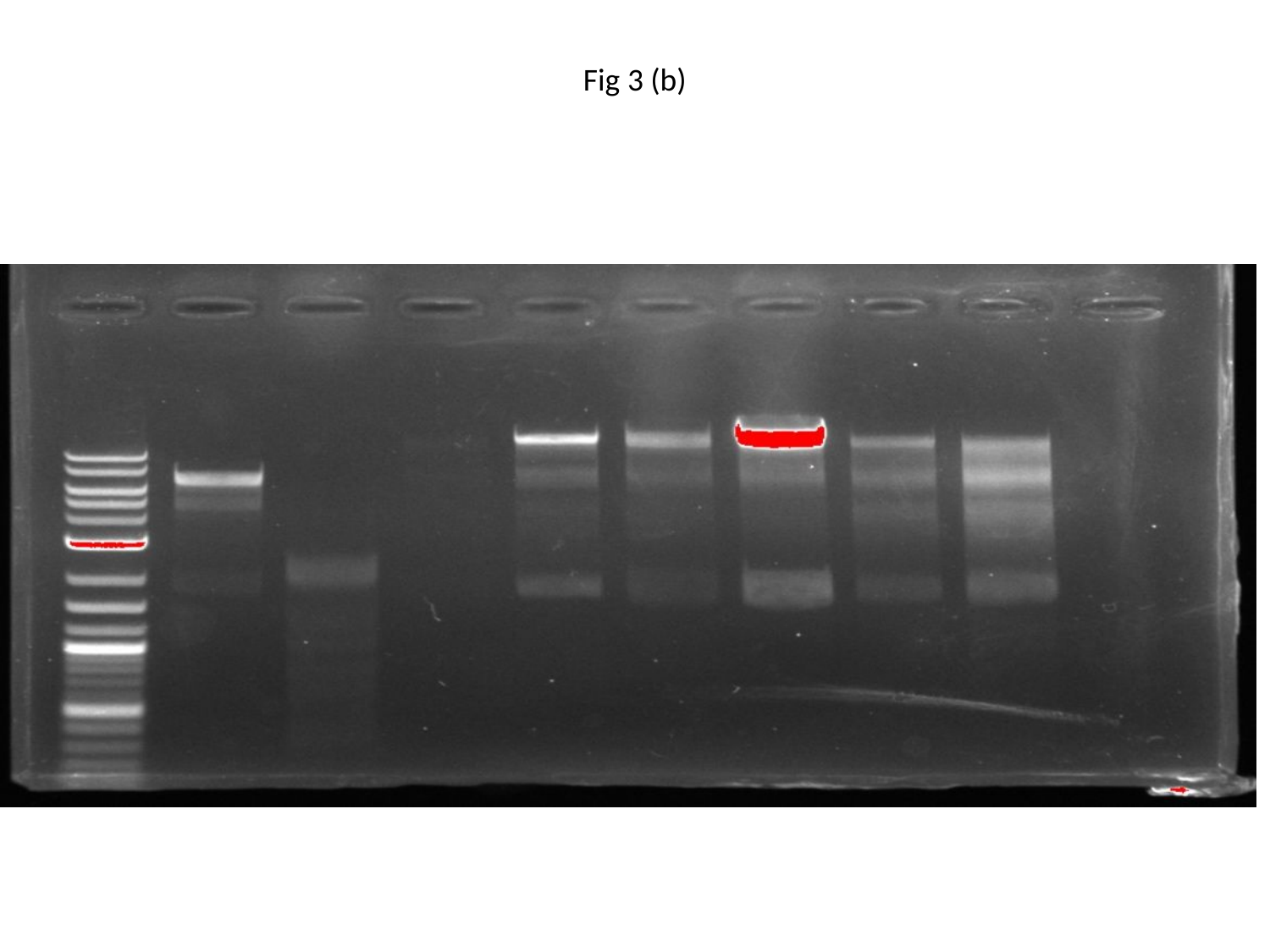

Fig 3 (b)

## Slide 2
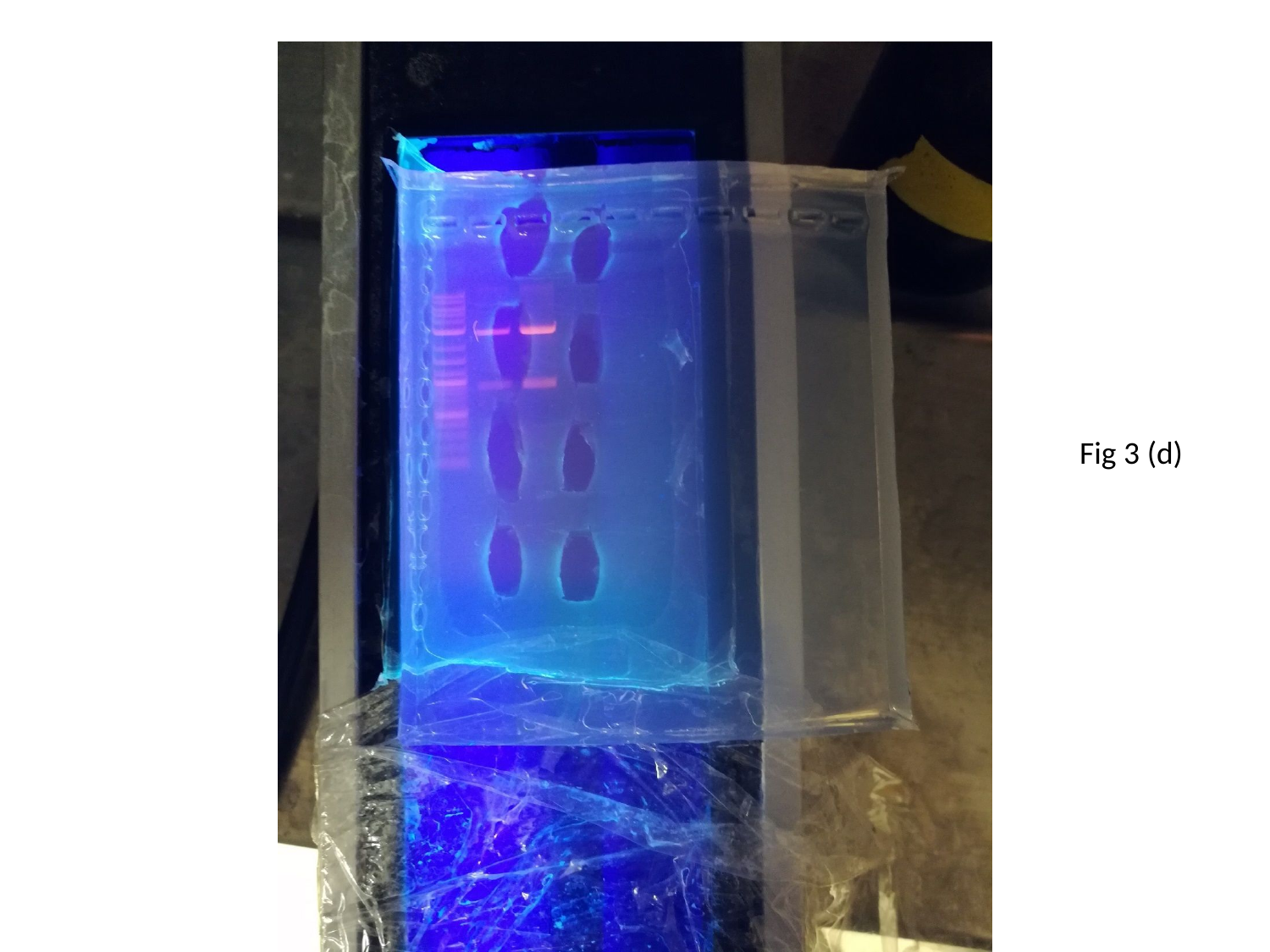

Fig 3 (d)

## Slide 3
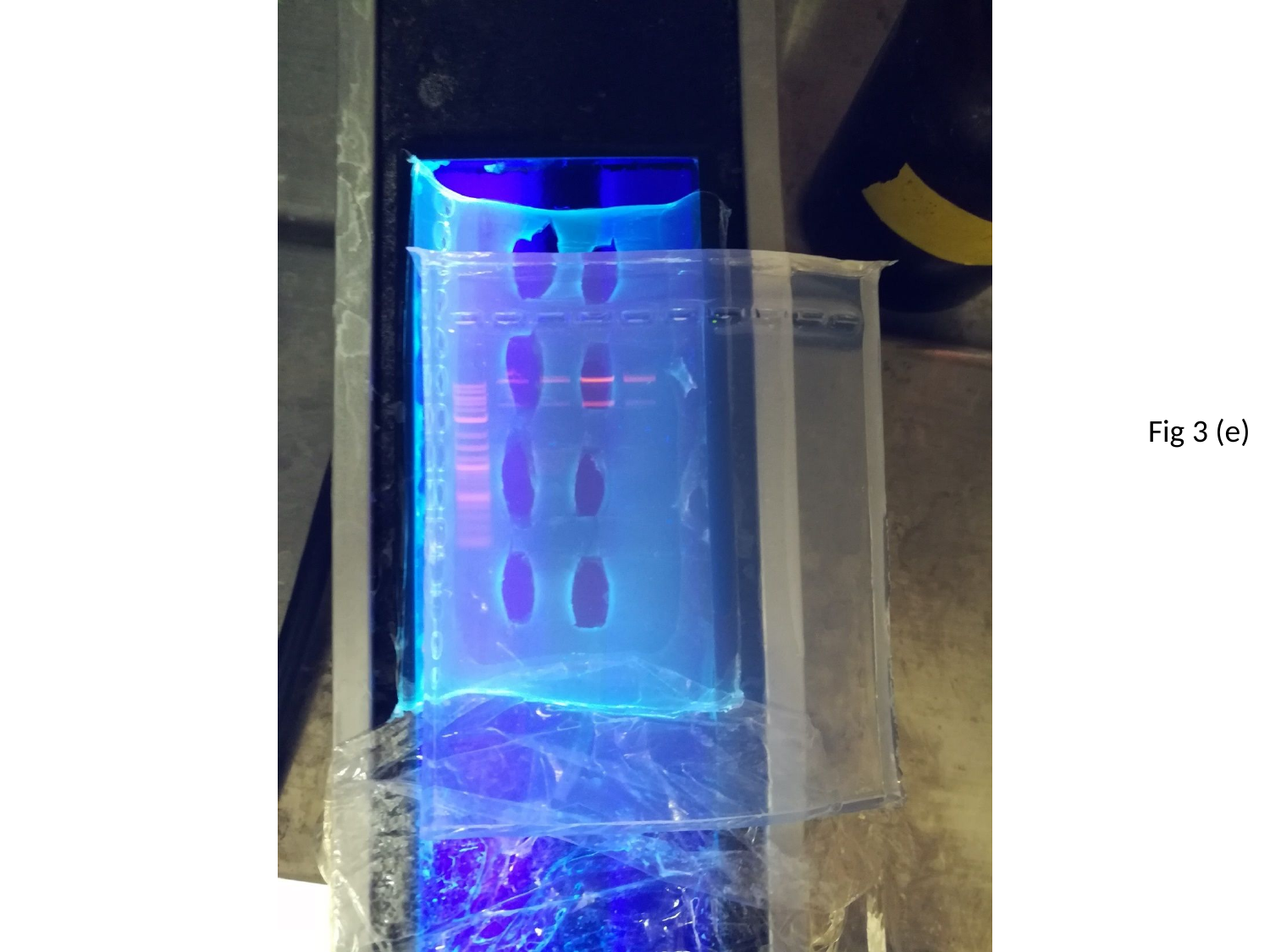

Fig 3 (e)

## Slide 4
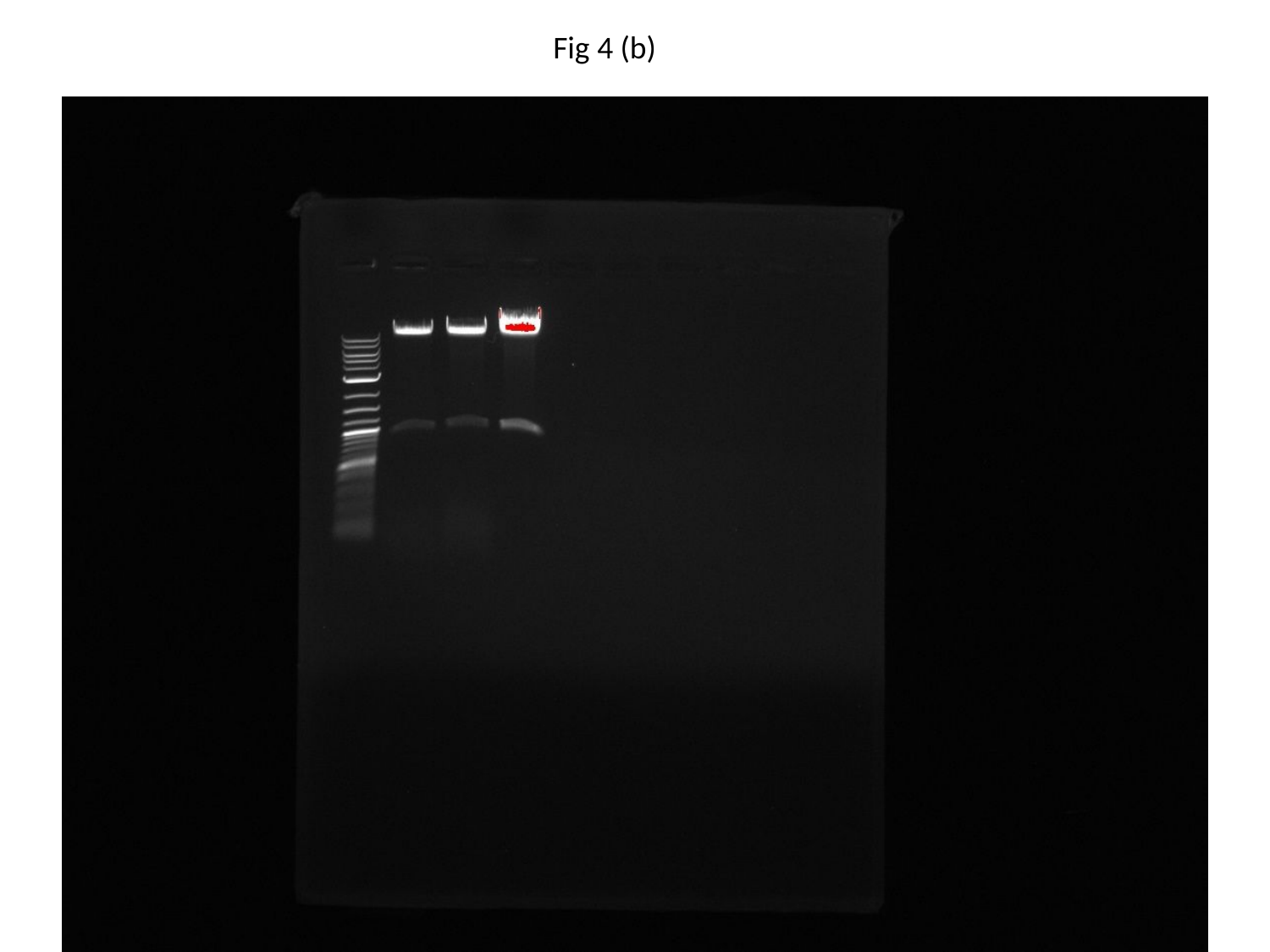

Fig 4 (b)

## Slide 5
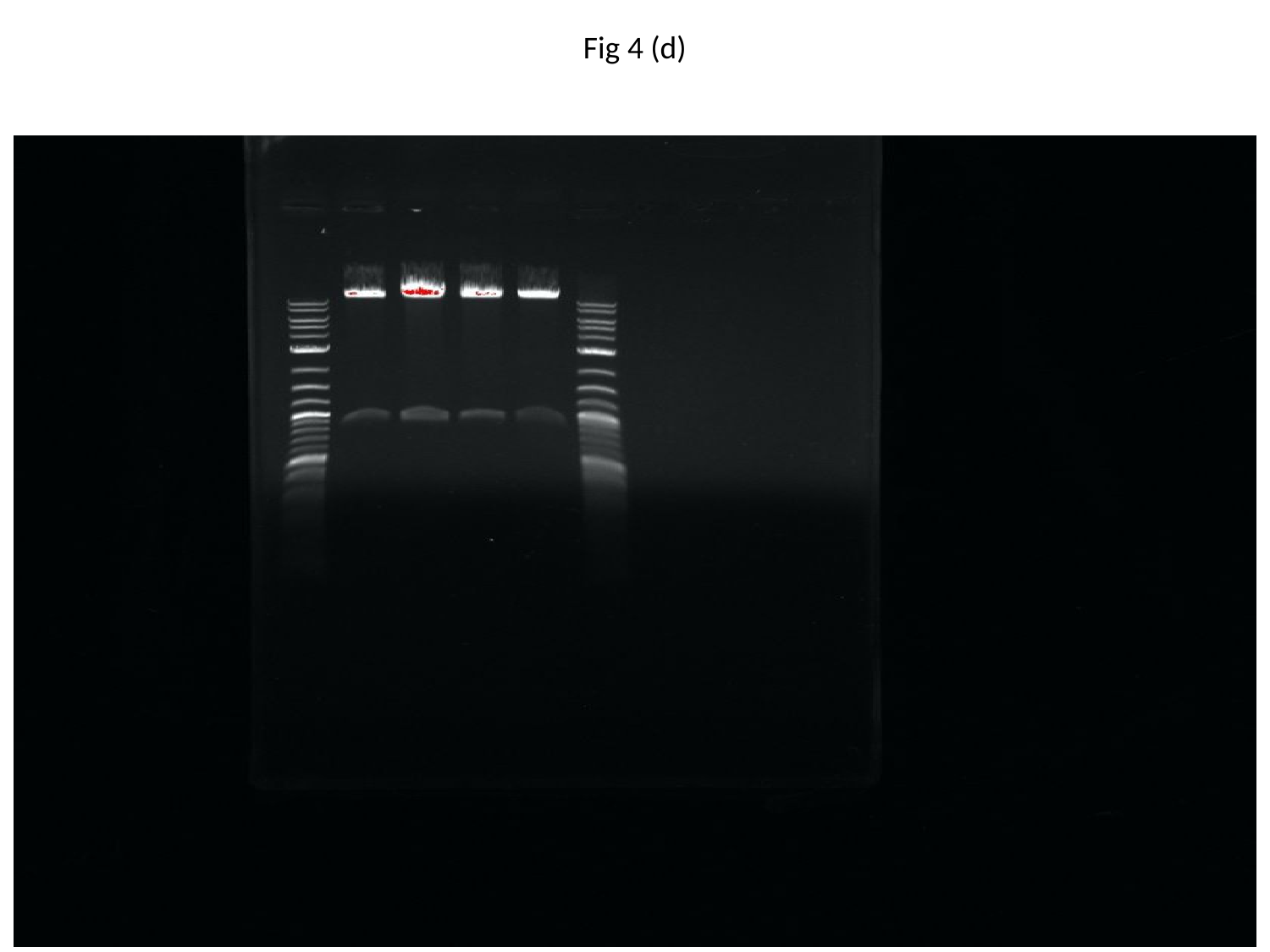

Fig 4 (d)

## Slide 6
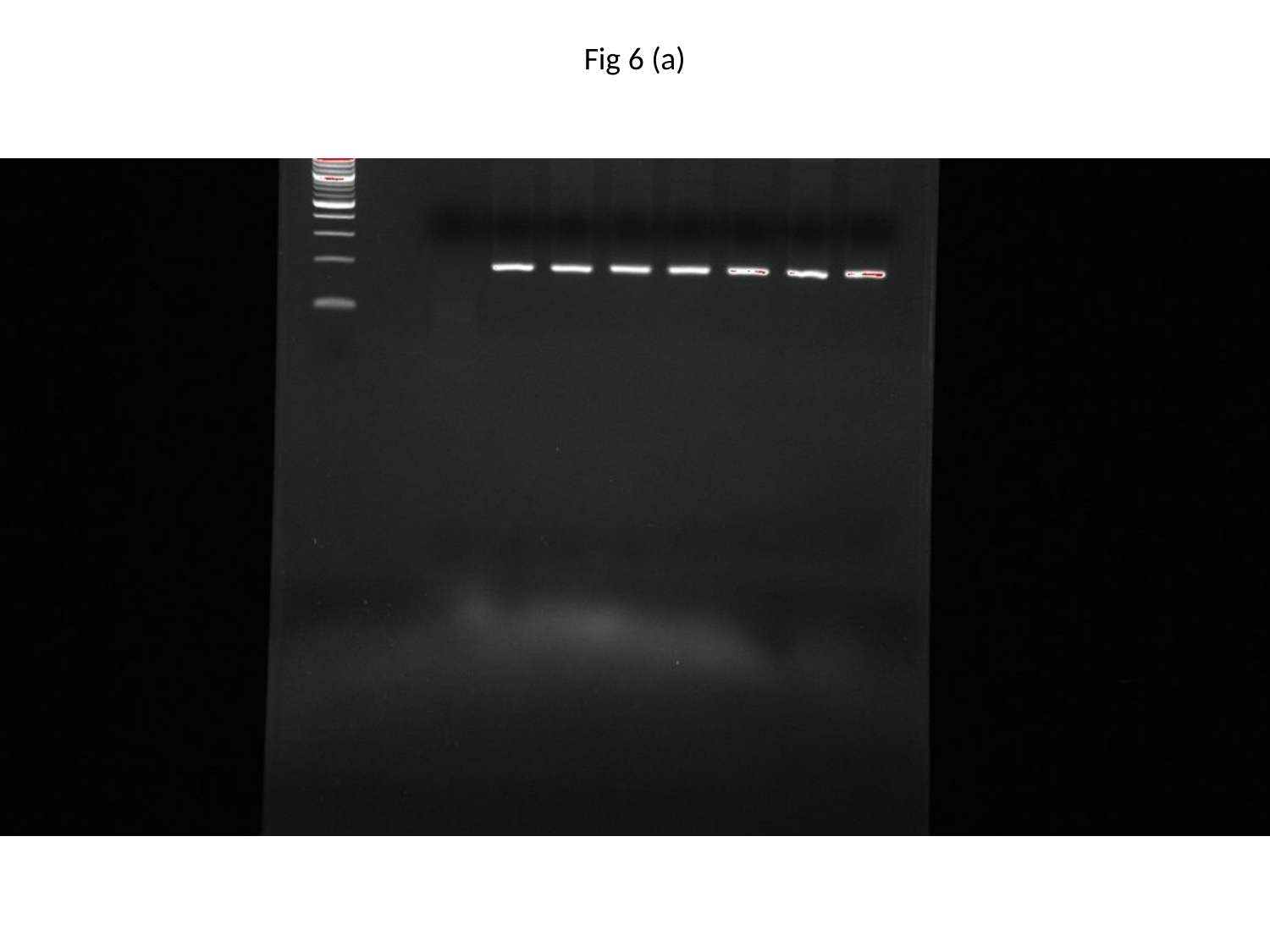

Fig 6 (a)

## Slide 7
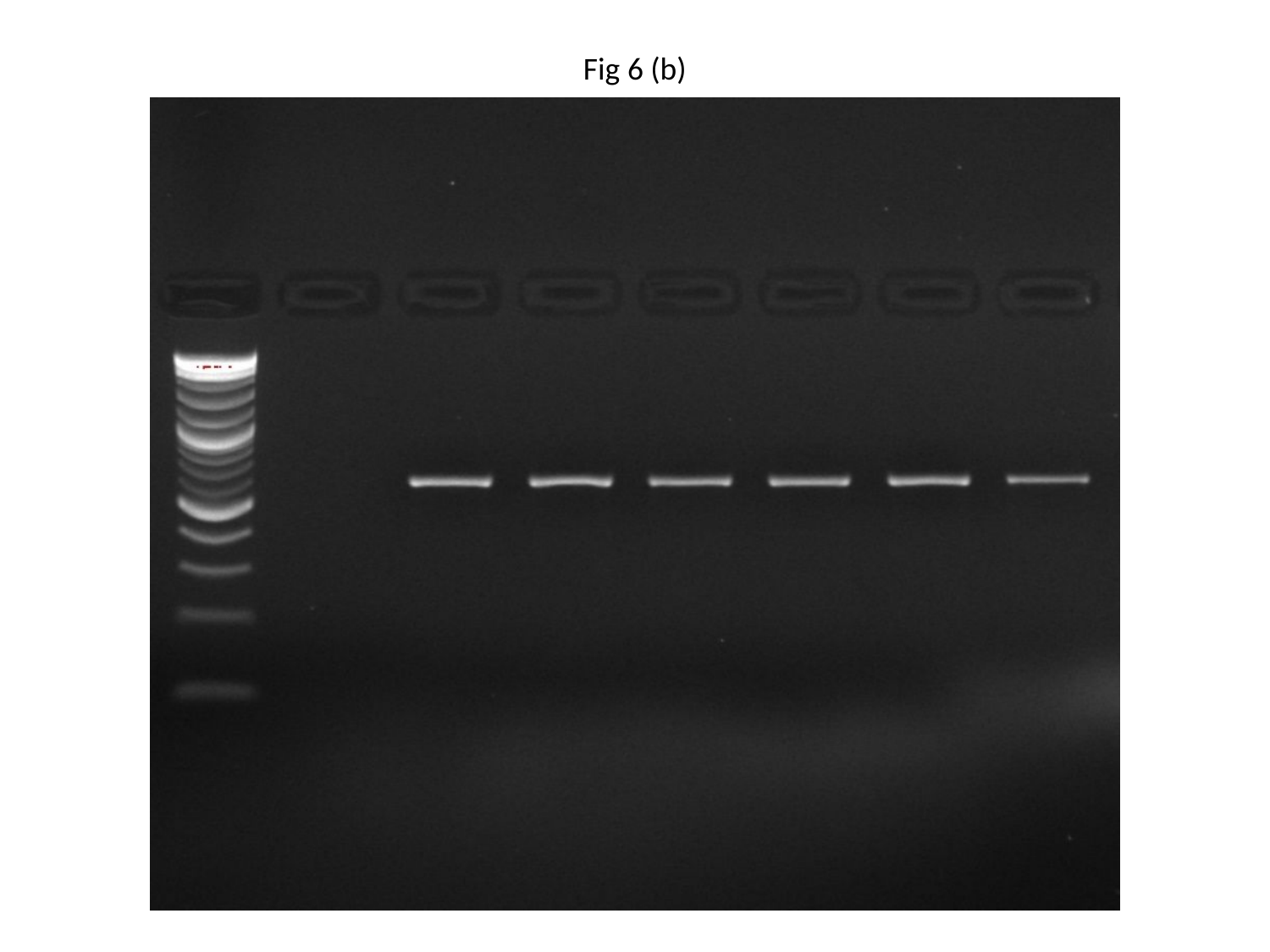

Fig 6 (b)

## Slide 8
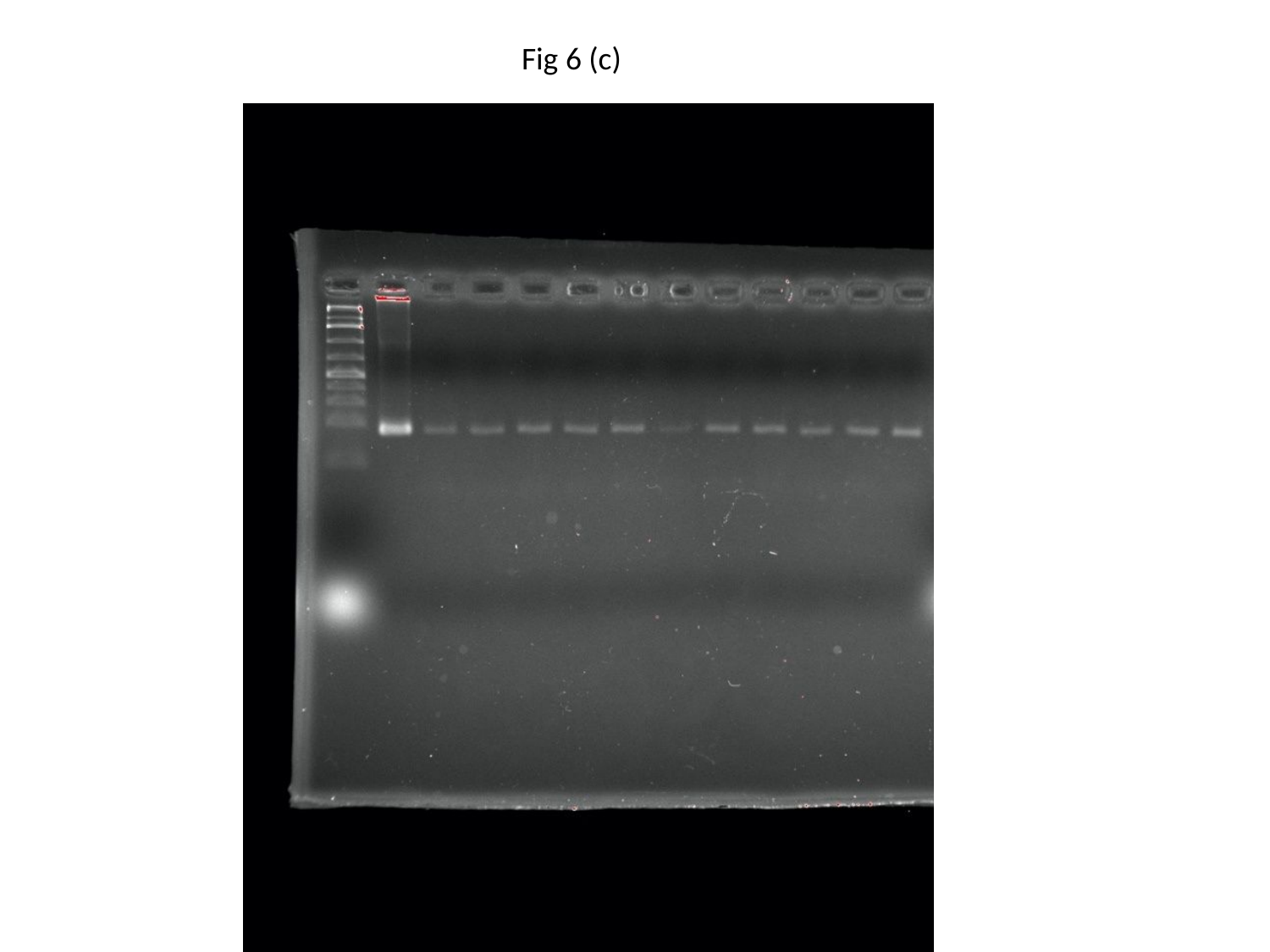

Fig 6 (c)

## Slide 9
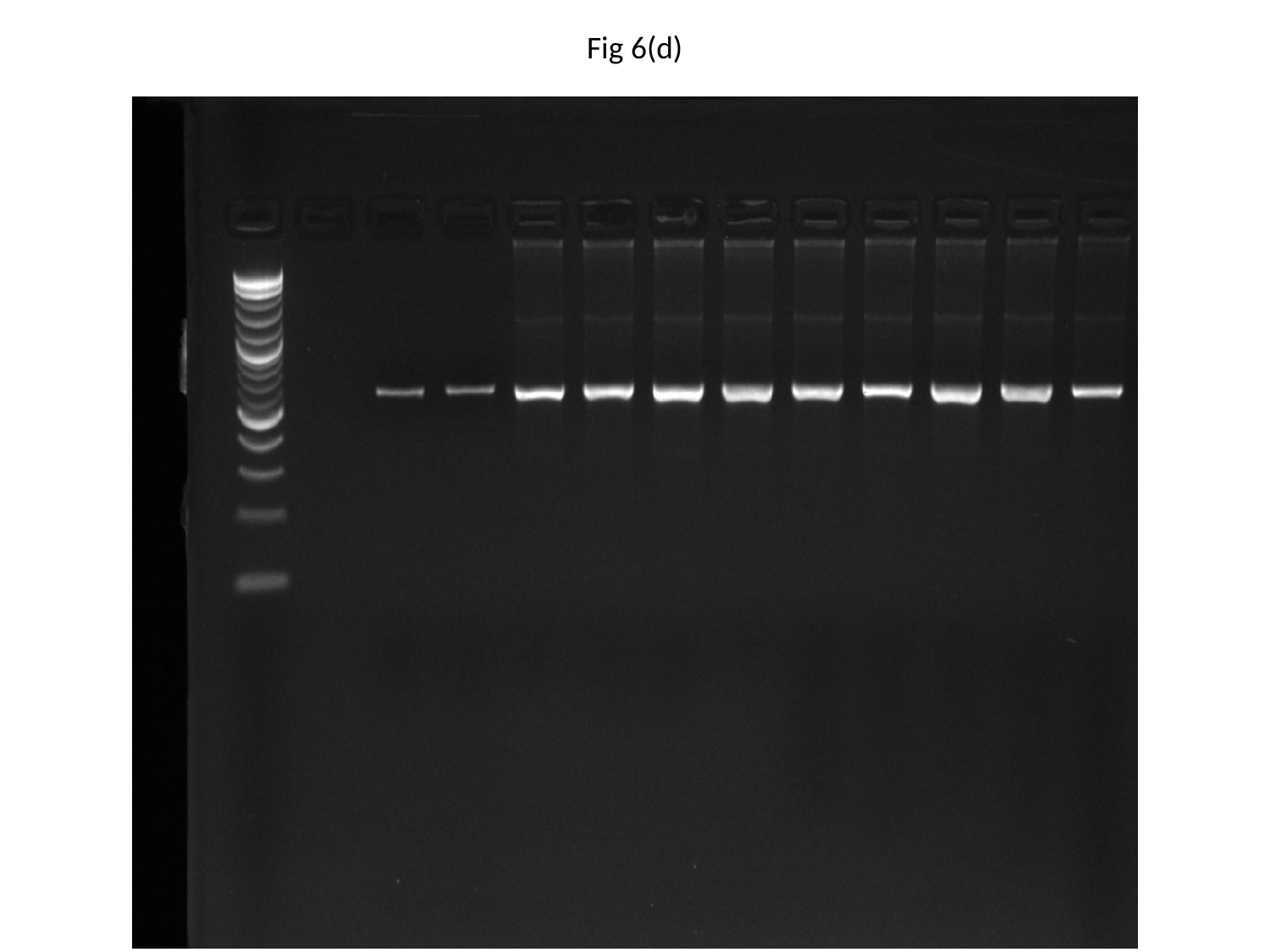

Fig 6(d)
