# Supplementary material for: Cloning of maize chitinase 1 gene and its expression in genetically transformed rice to confer resistance against rice blast caused by Pyricularia oryzae
Source: PLoS One. 2024 Jan 16;19(1):e0291939. doi: 10.1371/journal.pone.0291939 (PMC10791007; doi:10.1371/journal.pone.0291939)
Supplement: Attachment — Submitted filename: Response to Comments.docx [file pone.0291939.s003.docx]

**Response to Academic Editor:**

**Please give pointwise replies to the comments raised by the reviewers**

Answer: All comments are replied

**Please ensure to have the language editing and check for typo errors**

Answer: Manuscript has been revised and proofread completely to remove all language, grammar and typing errors.

**Discussion section is invariable need to improve**

Answer: Discussion part has been revised completely as per reviewers guidelines

**Response to Journal Requirements:**

**1. Please ensure that your manuscript meets PLOS ONE's style requirements, including those for file naming. The PLOS ONE style templates can be found at**

Answer: Changes has been incorporated.

**2. We note that the grant information you provided in the ‘Funding Information’ and ‘Financial Disclosure’ sections do not match. When you resubmit, please ensure that you provide the correct grant numbers for the awards you received for your study in the ‘Funding Information’ section.**

Answer: Please add the following grant number “HEC-NRPU-1854”

**3. Thank you for stating the following in the Acknowledgments Section of your manuscript:**

Answer: Please update our Funding Statement as following

“We are thankful to Higher Education Commission Pakistan, for providing funding to conduct the current research under grant number HEC-NRPU-1854. No additional external funding was received for this study. The funding agency had no role in study design, data collection and analysis, decision to publish, or preparation of the manuscript.”

**4. PLOS ONE now requires that authors provide the original uncropped and unadjusted images underlying all blot or gel results reported in a submission’s figures or Supporting Information files.** In your cover letter, please note whether your blot/gel image data are in Supporting Information or posted at a public data repository, provide the repository URL if relevant, and provide specific details as to which raw blot/gel images, if any, are not available. Email us at [plosone@plos.org](mailto:plosone@plos.org) if you have any questions.

Answer: Please add the data as supporting information.

**5. Please amend your list of authors on the manuscript to ensure that each author is linked to an affiliation. Authors’ affiliations should reflect the institution where the work was done (if authors moved subsequently, you can also list the new affiliation stating “current affiliation:….” as necessary).**

Answer: List of authors is linked with affiliation.

**Response to Reviewers Comments**

| **Reviewer #1** | |
| --- | --- |
| 1. Language editing and check for typo errors is needed. | Manuscript has been revised and proofread completely to remove all such errors. |
| 2. In line 122: are pSDF1 and pDW3586 same vectors? | Yes, you are right this is the same vector and it has been explained in revised manuscript. |
| 3. Line 133-134: Whether pSDF1 and pSDF2 plasmids are of same size? How can you get 10 kb and 0.9 kb DNA size in both the vectors when restriction digested with SpeI and Bam HI? pSDF1 is pDW3586 while pSDF2 is pUC57 with MC1. | 10Kb fragment was produced after restriction digestion of pSDF1 while 0.9Kb fragment was produced after restriction digestion of pSDF2. Statement has been corrected in the revised manuscript. |
| 4. Please write the correct name of Agrobacterium strain. It is EHA 101 not the EH 101. | Corrections has been made in the revised manuscript. |
| 5. Fig. 2: Please show properly the T-DNA region. All components should be within LB and RB regions. | Changes has been incorporated in the revised figure. |
| 6. Line 185: Is it table 1 or 3.6 | Its table 1. Thanks for pointing out. Correction has been made in the revised manuscript. |
| 7. In Table 1, for infection formulation pH 7.0 was used which is not common for Agrobacterium transformation experiments. pH 5.2 is usually preferred. | Yes, you are right, in our experiments, pH was optimized as 5.8 and correction has been made in the revised manuscript. |
| 8. Line 216-220. Denaturation in PCR at 72oC is not correct. Please verify. | Correction has been made in the revised manuscript. |
| 9. Line 260-262: Whether plasmid pDW3586 has two sizes- 13.5 kb and 4 kb? In D1-D3 lanes 9.1 kb and 6 kb bands were inappropriate. Moreover, in D2, D3 lanes bands are absent. Mostly the gel images are not matching with the text given in results. Provide an appropriate gel image (3b). | It was typing mistake. The actual size is 17.34kb. Correction has been made in the manuscript. D1, D2 and D3 represents different time of restriction incubation and has been explained in the revised manuscript. |
| 10. Fig5. Replace with good image. Also add the rooting and hardening stage images. | Changes has been incorporated as suggested. |
| 11. In table 7, just mention as T0 or T1 lines. No need to write name of rice variety. | Changes has been incorporated as suggested. |
| 12. Line 419: Some text is missing in leaf detachment assay | Correction has been made in the revised manuscript. |
| 13. Whole plant assay must be conducted along with leaf detachment assay and the results should be included. | Thank you for your positive suggestion. Infect due to limitation of T1 generation plants, we only focused on the leaf detachment assay. We used leaf detachment assay because in literature it has been used as standard assay in multiple studies. |
| 14. Lines 424-427: Sentences are repeated from abstract and introduction sections. | Statements has been replaced in the revised version. |
| 15. Discussion should be improved a lot. Nowhere in the entire manuscript was mentioned regarding the significance of blast disease. What is the importance of this work? Most of the work is w.r.t. standardizing the protocol for Basmati variety. The discussion should have focused on chitinase gene and blast disease. | Discussion has been revised completely as per suggestions. |
| 16. In the sentences such as “High gene expression level was explained by [69]”, authors name should be written along with the number for citation. | Changes has been incorporated in the revised manuscript. |
| 17. Instead of using the term parent vector, please use standard terms such as cloning vector, expression vector and binary vectors or simply a vector. | Changes has been incorporated in the revised manuscript. |
| **Reviewer #2:** | |
| 1. The article is of scientific interest but the article needs thorough revision. It is better to get it edited using an expert in English language. The MS is too elaborative. | Manuscript has been revised and proofread completely to remove all such errors. |
| 2. Fig-7 does not represent the differences of control and transgenic plants inoculated with P. oryzae. The Figure 7(a) it seems all the plants are suffering from nutrient deficiency. Similarly, please check the Fig7. (b) there are huge differences among all the three plants as per as the nutrient supply is concerned. | Thank you for your suggestion. Figure 7 represents the relative gene expression of MC1 gene in T0 and T1 generation which was quantified by real time PCR. The difference in bar represents the gene expression relative to control |
| 3. The first criteria of blast infection is the application of heavy dose of nitrogen to the plant which is not seen in your experiment. | Thank you for your insight review. Infect our main objective of the research was cloning of MC1 gene and its expression analysis so we limited our applications only on transgenic line in comparison of non-transgenic lines. |
| 4. The species of Agrobacterium is not mentioned anywhere. | Specie information has been added in the revised manuscript. |
| 5. In line 233 the authors have written "Total RNA extraction was done by method reported by [84] with slight modification": this type of sentence should be avoided. The sentence should be written as "reported by Liu [et.al](http://et.al/). [84]". | Changes has been incorporated in the revised manuscript. |
| 6. Kindly modify the MS thoroughly checking even the technicality and the language. | Manuscript has been revised completely to remove all such errors. |
| 7. In Fig 3 why you have used two different backgrounds for the gel photography of 3.b & d? | Thank you for your time and positive feedback on our manuscript. The gel images were observed using two different instruments and we intended to provide the original images in the manuscript that’s why we added them as it. |
