# Supplementary material for: Cloning of maize chitinase 1 gene and its expression in genetically transformed rice to confer resistance against rice blast caused by Pyricularia oryzae
Source: PLoS One. 2024 Jan 16;19(1):e0291939. doi: 10.1371/journal.pone.0291939 (PMC10791007; doi:10.1371/journal.pone.0291939)
Supplement: Attachment — Submitted filename: Authors reponse to reviewer comments.docx [file pone.0291939.s002.docx]

**Review Report (Plos one)**

**Reviewer 1**

| **Editorial Comments** | **Authors Response** |
| --- | --- |
| 1.  Please upload a new copy of Figure 1 as the detail is not clear. Please follow the link for more information: | We have removed Fig. 1 because same information is provided in the tables. |
| 2. It appears that you did not upload a Track Changes version of your manuscript file, which highlights any changes you made to your previous submission (PONE-D-21-17119). | Manuscript with tack changes have been uploaded in the journal submission system. |
| **Reviewers Comments** | **Authors Response** |
| 1. Line 121-122: ‘The parent plasmid was modified by digesting it with Xho I /Xba I and annealing phosphorylated oligos at both ends; the modified plasmid was renamed pSDF 1 (Fig 1).’  It is not understood why the authors need to anneal oligos. | The parent vector pDW 3586 was cut with endonucleases pair, synthetic and phosphorylated oligos were aligned at both the ends. This created sticky ends that facilitated *MC*1 gene integration. |
| **2.** This vector is a vector for genome editing that contains Cas9 and gRNA expression cassettes.  Line 163: ‘The details of tDNA region of vector used in the study is given in (Fig 2)’.  Figure 2 is wrong. The tDNA portion contains Cas9 and gRNA expression cassette. So, it is a genome editing vector. I do not see anywhere the mention of Chitinase gene. | The vector was used for CRISPR expression in rice as well (Banakar *et al.,* 2019). The detailed vector map of pDW 3586 is presented in the given figure. The T-DNA has rice U6 promoter #1, partial sgRNA and terminator, XbaI, EcoRI, modified maize UBI Promoter, Maize chitinase 1 gene, 35s terminator, Spectinomycin resistance gene, Left border, Nos terminator, hygromycin gene, 2x35s promoter.  The rice CAS9 gene was removed and MC1 gene was inserted at its place.  Corrected figure of T-DNA region used in the current research.   |
| Table 6 Transformation frequency of hygromycin resistant calli on regeneration medium’ Incorrect | Percentage can be calculated by dividing the value by the total value, and then multiplying the result by 100. The formula used to calculate percentage is: (value/total value)×100%.  So according to the formula the calculated percentages were correct. |
| Table 7 Functional validation of transgenic plants by leaf detached assay’  Non transgenic lesion size is given as 89 cm. Which is absurd. | In table 7 the lesion size of Non transgenic plant was recorded 8.66 cm, 89 was a typing error. |
| Only detached leaf bioassay is not sufficient to back the claim that the transgenic plants are resistant against blast. Bioassay picture is not provided. Whole plant bioassay need to be done. |   Figure (a) **Photographic representation of transgenic plants at T0 generation**  a: Control plant infected with *P. oryzae*  b: Transgenic plant before infection with *P. oryzae*  c: Transgenic plant before infection *P. oryzae*    Figure (b) **Photographic representation of transgenic plants at T1 generation**  a: Control plant infected with *P. oryzae*  b: Transgenic plant before infection with *P. oryzae*  c: Transgenic plant before infection *P. oryzae* |
| Line 555-556: “In addition, the expression of the *MC1* displayed no harmful effects on the transgenic plants, which are consistent with the results that chitinases are not toxic to host plant” Not backed by any data. | The figures (a) and (b) above are the transgenic plants and the control plants which is a clear proof that as compared to non- transgenic plant, the transgenic plants at both the generations showed improved growth. Moreover, height of plants, number of leaves, number of tillers produced were also recorded but due to the length of the paper these were not included in the main manuscript. |

**Reviewer Report (Plos One)**

**Report 2**

| **Reviewers Comments** | **Authors Response** |
| --- | --- |
| 1.Molecular characterization of transgenic plants: in this article molecular analysis was  carried out by PCR method. The problem is that PCR analysis will not provide information on stable integration of transgene and number of copies of transgene in the genome. In order to confirm the transgenic status of rice plants the Southern blot analysis is essential. | For stable integration of transgene and number of copies of transgene in the genome, qPCR for transgene number was performed and DNA copy number was calculated using the following formula:  **Calculations: DNA copy Number for qPCR**  *Formula used: Amount of DNA (ng)x 6.022x10^23^*  *____________________________*  *Length (bp)x 1x 10^9^x 660*  Where Amount of DNA in 262 ngs  6.022x10^23^ is Avogadro’s Number  Length of template DNA is 903 bps  1x 10^9^ is the conversion factor  660 is the average mass of 1bp dsDNA.  By Putting values in the above equation:    262x 6.022x10^23^  _______________ = 2.6x10^11^    903x 1x10^9^ x 660  Moreover, segregation analysis on seed from the primary transgenic plants was performed to show transmission of the transgene to get some idea of copy number. |
| 2. For expression analysis of chitinase gene, the authors adopted RT-PCR method. As shown in Fig 7, it is evident that there is no clear pattern of chitinase gene expression in T0 and T1 plants. In this case, Western blot analysis is required to confirm the results of RT-PCR. | qPCR analysis was performed to check the expression of chitinase gene at both T0 and T1 Actin was utilized as internal control for data standardization. The comparative quantification of gene expression was analyzed by using delta Ct method in tested samples and standard deviation was calculated.The graphs were plotted for relative expression of *MC1* gene as compared to the internal control clearly demonstrated the high expression of transgene as compared to internal control. |
| 3.For evaluation of blast resistance, the authors should provide in planta evaluation data without that the results are not justifiable. |   Figure (a) **Photographic representation of transgenic plants at T0 generation**  a: Control plant infected with *P. oryzae*  b: Transgenic plant before infection with *P. oryzae*  c: Transgenic plant before infection *P. oryzae*    Figure (b) **Photographic representation of transgenic plants at T1 generation**  a: Control plant infected with *P. oryzae*  b: Transgenic plant before infection with *P. oryzae*  c: Transgenic plant before infection *P. oryzae* |
