# Supplementary material for: Cloning of maize chitinase 1 gene and its expression in genetically transformed rice to confer resistance against rice blast caused by Pyricularia oryzae
Source: PLoS One. 2024 Jan 16;19(1):e0291939. doi: 10.1371/journal.pone.0291939 (PMC10791007; doi:10.1371/journal.pone.0291939)
Supplement: Attachment — Submitted filename: Author response to reviewer comments.doc [file pone.0291939.s004.doc]

**Journal Requirements:

Please review your reference list to ensure that it is complete and correct. If you have cited papers that have been retracted, please include the rationale for doing so in the manuscript text or remove these references and replace them with relevant current references. Any changes to the reference list should be mentioned in the rebuttal letter that accompanies your revised manuscript. If you need to cite a retracted article, indicate the article’s retracted status in the References list and also include a citation and full reference for the retraction notice.**

Answer: All references has been cross checked and corrections has been made in the revised version **Additional Editor Comments:

Authors have addressed the concerns raised by the reviewers. But I suggest authors to improve the language of the paper by taking help of the professional English editing services.**

Answer: Manuscript has been revised and proofread completely by American native language speaking person (Letter attached) to remove all language, grammar and typing errors.

**The use of mycelial plugs from 48 hr old culture to inoculate for testing disease resistance need to substantiate...**

Answer: We agree with the reviewer comments and experiment validation has been added in the revised version.

**Review Comments to the Author**

**Reviewer #3: The authors have incorporated all the modifications as suggested. But till then I suggest please check the spellings once more.**

Answer: Thank you for your time and feedback. Manuscript has been revised and proofread completely to remove all language, grammar and typing errors.

**Reviewer #4: I have gone through the comments of earlier reviewers. While the authors have tried to address the concerns raised by the reviewers, I still believe that the authors should improve the language of the paper by taking help of the professional English editing services.**

Answer: Manuscript has been revised and proofread completely to remove all language, grammar and typing errors.

**I have noted a gross methodological error committed by the authors while screening the resistance of the trasngenics expressing the chitinase gene. The use of the mycelial plugs from 48 hr old culture to inoculate rice leaves for testing their resistance is absolutely incorrect because the mycelial strands cannot penetrate and infect the aerial plant surfaces of the rice. Probably the authors are not aware about the infection biology of the blast pathogen. The infection of rice leaves invariably occurs through the conidial inoculum, which is applied to the leaves as spore suspension or as spore droplets. Infection begins when spores germinate on the leaf surface and form appressoria at the germ tube tips. Appressoria become pressurized and melanized. When tightly annealed, hydrostatic turgor acts on a penetration peg at the appressorial base, forcing it to penetrate the cuticle. It is therefore necessary to apply a conidial suspension to leaves to evaluate their resistance to blast. I am providing the references of a few papers, which report on evaluation on the resistance of transgenics expressing chitinase or other defence response genes against rice blast; in all these studies conidial suspensions have been applied to test the resistance of rice plants against leaf blast.**

Answer: Yes, you are right. We also used the spores suspension for testing the disease resistance. Initially we added the summarized version of methodologically in which we unintendedly, missed some important steps. Thanks for your insightful review to highlight the importance and mechanism of the bioassay. The detail of methodology has been added in the revised manuscript. This revised methodology is SOP of our laboratory, as in one of the previous studies we also utilized the same methodology in which spores were utilized.

ANWAAR, SADAF, et al. "EVALUATION OF PAKISTANI RICE CULTIVARS FOR APPARENT INFECTION RATE AGAINST FUNGAL PATHOGEN Pyricularia oryzae." *PLANT CELL BIOTECHNOLOGY AND MOLECULAR BIOLOGY* (2021): 29-37.
