# Supplementary material for: Cloning of maize chitinase 1 gene and its expression in genetically transformed rice to confer resistance against rice blast caused by Pyricularia oryzae
Source: PLoS One. 2024 Jan 16;19(1):e0291939. doi: 10.1371/journal.pone.0291939 (PMC10791007; doi:10.1371/journal.pone.0291939)
Supplement: Attachment — Submitted filename: Respone to reviewer comments.docx [file pone.0291939.s005.docx]

**Journal Requirements:**

**Please review your reference list to ensure that it is complete and correct. If you have cited papers that have been retracted, please include the rationale for doing so in the manuscript text or remove these references and replace them with relevant current references. Any changes to the reference list should be mentioned in the rebuttal letter that accompanies your revised manuscript. If you need to cite a retracted article, indicate the article’s retracted status in the References list and also include a citation and full reference for the retraction notice.**

Answer: Reference list has been checked carefully, it is complete and moreover no paper has been cited which is retracted.

**Additional Editor Comments:**

**The article is of scientific interest, but the article needs still revision.**

**It is better to get it edited using an expert in English language.**

**The manuscript is too elaborative. Figures need to be revised. Discussion needs to be improved a lot. Please go through several comments of reviewers and reply suitably**

Answer: Manuscript has been revised

**Review Comments to the Author**

**Reviewer #3: The authors have answered all the queries asked by the reviewers. My only concern is about the quality of photographs especially the gel photographs.**

Answer: Thank you for your time and feedback. Quality of manuscript has been improved in the revised manuscript.

**Reviewer #4: As has been the case with many of the queries raised by other reviewers, in the instant case also the authors knowing well that they have committed a fatal methodological error in screening of their transgenic lines, they have changed their stance and now claiming that they have the used the other screening method suggested by me to screen the transgenic lines. Their assertion is unbelievable and cannot be trusted.**

Answer: Thank you for your time and concern regarding methodology. Initially, revision was requested on leaf detached assay and we are thankful for the reviewer for identifying missing information. We have just added the missing information and provided the complete methodology. Additionally, we would like to add that using spore suspension is a routine methodology of our laboratory and it is our standardized SOP which can also be confirmed from our other publication “ANWAAR, SADAF, et al. "EVALUATION OF PAKISTANI RICE CULTIVARS FOR APPARENT INFECTION RATE AGAINST FUNGAL PATHOGEN Pyricularia oryzae." *PLANT CELL BIOTECHNOLOGY AND MOLECULAR BIOLOGY* (2021): 29-37.” Moreover, the reference we cited in the relevant section also support our methodology for using spore suspension. We just used the summary of methodology and cited the reference which ultimately means that we have followed the same method as mentioned in the reference.
